# The correlation of left atrial diameter with preserved ejection fraction, reduced ejection fraction, and mid‐range ejection fraction

**DOI:** 10.1002/clc.24134

**Published:** 2023-08-25

**Authors:** Jing Lin, Huajui Wu, Tianwen Zhang

**Affiliations:** ^1^ Department of Cardiology Ningbo Medical Center Lihuili Hospital Ningbo City China; ^2^ Ningbo Aier Guangming Eye Hospital Ningbo City China

**Keywords:** left atrial diameter, preserved ejection fraction, reduced ejection fraction and mid‐range ejection fraction

## Abstract

**Background:**

In patients with heart failure, left atrial remodeling often occurs to varying degrees. Left atrial enlargement has been proved to be an important predictor of cardiovascular‐related adverse events. However, the relationship between left atrial diameter (LAD) with heart failure (HF) with preserved ejection fraction (HFpEF), reduced ejection fraction (HFrEF) and mid‐range ejection fraction (HFmrEF) remains unclear.

**Hypothesis:**

We want to explore the correlation between left atrial diameter and HFpEF, HFmrEF, and HFrEF.

**Methods:**

A total of 210 patients with heart failure who underwent hospitalization in Ningbo Medical Center Lihuili Hospital, Zhejiang, China, from January 1, 2020, to June 31, 2021, were reviewed. The basic demographic characteristics, blood test, and the related indexes of echocardiography of the subjects were collected and analyzed.

**Results:**

There is a significant difference between HFpEF and HFrEF group in LAD (*p* = .007), and LAD is negatively correlated with left ventricular ejection fraction (LVEF) (*p* = .002, *r* = −.209).

**Conclusion:**

LAD is negatively correlated with LVEF, which may predict the prevalence of HFrEF.

## Graphical abstract

We studied the data of 210 individuals with heart failure. There is a significant difference between HFpEF and HFrEF groups in LAD (*p* = .007). And LAD is negatively correlated with LVEF (*p* = .002, *r* = −.209), which may predict the prevalence of HFrEF.

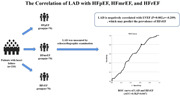

## 1 INTRODUCTION

The 2016 ESC guidelines divide heart failure into three subgroups according to left ventricular ejection fraction (LVEF): heart failure reduced ejection fraction (HFrEF), heart failure preserved ejection fraction (HFpEF), and heart failure with mid‐range ejection fraction (HFmrEF).^1^ Recent studies have found that HFmrEF is different from traditional HFrEF and HFpEF in clinical manifestations, laboratory examination, and prognosis, and can be regarded as an independent type.^2^, ^3^ Some studies also suggest that HFmrEF is a transitional stage or overlapping region between HFrEF and HFpEF, rather than a unique subtype of heart failure.^4^, ^5^ Further research is needed to better clarify the pathophysiology, treatment, and prognosis of HFmrEF.^6^

The left atrium plays a key role in regulating left ventricular filling and cardiovascular performance by reserving pulmonary venous return and augmenting ventricular filling.^7^ Different left ventricular remolding types were found in HFpEF, HFmrEF, and HFrEF patients.^8^, ^9^, ^10^, ^11^, ^12^ Left atrial enlargement has been proved to be an important predictor of cardiovascular‐related adverse events, such as atrial fibrillation, heart failure, and cardiovascular death.^13^

Although left atrial anteroposterior diameter (LAD‐ap) is not as accurate as left atrial volume in evaluating the volume of left atrium, LAD is simple, convenient, and can be used to predict the clinical outcome of heart failure.^14^, ^15^

However, the relationship between LAD with HFpEF, HFmrEF, and HFrEF remains unclear. The results showed that the LAD of HFmrEF was smaller than that of HFrEF and HFpEF.^2^ Some studies pointed out that the LAD of HFrEF was larger than that of HFmrEF and HFpEF, but there was no significant difference between HFmrEF and HFpEF.^16^ Therefore, this study retrospectively explored the correlation between left atrial diameter with HFpEF, HFmrEF, and HFrEF.

## 2 DATA AND METHODS

This research was conducted following the Declaration of Helsinki. This retrospective study was approved by the Institutional Review Board of Ningbo Medical Center Lihuili Hospital (No. LY2023YJZ059) and waived the individual consent for this analysis.

### 2.1 Research objects

A total of 210 consecutive subjects with HF referred to Ningbo Medical Center Lihuili Hospital from January 1, 2020, to June 31, 2021, were reviewed. According to the LVEF, there were 70 patients (45 male patients, 64.3%) in HFrEF group (LVEF < 40%), 70 patients (male 39 male patients, 55.7%) in HFmrEF group (LVEF = 40%–49%) and 70 patients (32 male patients, 45.7%) in HFpEF group (LVEF ≥ 50%). Inclusion criteria: (1) Patients with complete medical records during hospitalization; (2) Patients met the diagnostic criteria of HF according to ESC 2016: Guidelines for Diagnosis and Treatment of Acute and Chronic Heart Failure.^1^ Exclusion criteria: (1) Complicated with acute myocardial infarction in recent 3 months; (2) Complicated with severe valvular heart disease; (3) Complicated with pulmonary heart disease; (4) Complicated with autoimmune diseases, severe infections, severe kidney diseases, blood system diseases, tumors, and so on.

### 2.2 Methods

In the study, the basic demographic characteristics of the subjects were collected and analyzed, including gender, age, smoking, and drinking; Previous disease history: hypertension and diabetes.

Blood test results during hospitalization included B‐type brain natriuretic peptide (BNP), creatinine (Cr), albumin, triglyceride (TG), total cholesterol (TC), low‐density lipoprotein, high‐density lipoprotein, and homocysteine (HCY).

The related indexes of echocardiography include left atrial diameter, left ventricular end‐diastolic diameter (LVEDd), left ventricular end‐systolic diameter (LVEDs), interventricular septum thickness at end‐diastolic (IVSd), left ventricular ejection fraction (LVEF) and left ventricular posterior wall diastolic thickness (LVPWD).

The medication of each person was counted, which was limited to diuretics, spironolactone, digoxin, anticoagulants, statins, β receptor blockers, angiotensin‐converting enzyme inhibitor (ACEI)/angiotensin‐receptor blocker (ARB).

### 2.3 Statistical methods

SPSS23.0 statistical software was used to test the distribution characteristics of samples with Shapiro–Wilk. The measurement data of normal distribution was expressed by mean ± standard deviation, and one‐way analysis of variance was used for comparison between groups. The measurement data of nonnormal distribution are expressed by median and quarterback difference, and independent sample median test and Bonferroni correction are adopted. The counting data were expressed as percentage, and the comparison between groups was carried out by two tests. Spearman's correlation coefficient was used to evaluate the correlation between left atrial diameter and HFpEF, HFmrEF, and HFrEF.

A receiver operating characteristic curve (ROC) was generated to assess the ability of the LAD to predict the prevalence of HFrEF and atrial fibrillation, and area under the curve (AUC) was calculated. The optimal cutoff value for the LAD was determined with the highest Youden index. A *p* < .05 was considered statistically significant.

## 3 RESULTS

### 3.1 Comparison of general data and auxiliary examination indicators

A total of 210 patients were included in the study, including HFpEF group, HFmrEF group, and HFrEF group, with 70 cases each. The proportion of basic diseases in HFpEF group was the highest (74.3%), followed by HFmrEF group (57.1%) and HFrEF group (35.7%) (*p* < .001). There was no significant difference in age, gender, smoking history, drinking history, diabetes history and atrial fibrillation among three groups (*p* = .146, *p* = .087, *p* = .114, *p* = .141, *p* = .152, and *p* = .175). There was no significant difference in BNP in HFpEF group was lower than that in HFrEF (547.50, 1160.25 vs. 1584.67, 2487.75, *p* < .001); BNP in HFpEF group was lower than that in HFmrEF group (547.50, 1160.25 vs. 1091.50, 1652.75, *p* = .021); There was no significant difference in BNP between HFrEF group and HFmrEF group (547.50, 1160.25 vs. 1091.50, 1652.75, *p* = .128).

LVEDd was the smallest in HFpEF group (48, 8.0), the second in HFmrEF group (56, 9.0) and the largest in HFrEF group (64, 11.25) (*p* < .001). Similarly, LVEDs in HFpEF group were the smallest (32, 8.0), followed by HFmrEF group (42, 9.25) and HFrEF group (54, 12) (*p* < .001). IVSd in HFpEF group was thicker than that in HFrEF group (11.4, 2.53 vs. 10.15, 2.23, *p* = .001), and IVSd in HFmrEF group was thicker than that in HFrEF group (11.2, 3.13 vs. 10.15, 2.23, *p* = .002). There was no significant difference in IVSd in HFpEF group and HFrEF group (11.4, 2.53 vs. 11.2, 3.13; *p* = 1.0). There was no significant difference in LVPWd among three groups (10.55, 2.5 vs. 10.2, 1.85 vs. 9.6, 1.4; *p* = .202).

In terms of biochemical indexes, there were no significant differences in Cr (77.5, 36.75 vs. 80, 35.25 vs. 100, 46.25; *p* = 1.0), albumin (36.25, 4.35 vs. 35.10, 5.93 vs. 35.70, 5.70; *p* = .313), TG (0.86, 0.61 vs. 0.92, 0.62 vs. 0.93, 0.52; *p* = .944), TC (3.42, 1.28 vs. 3.60, 1.13 vs. 3.47, 1.33; *p* = .476), HCY (11.85, 5.88 vs. 13.25, 6.13 vs. 16.5, 6.55; *p* = .876) among HFpEF, HFmrEF, and HFrEF.

In terms of drug treatment, HFmrEF and HFrEF groups used more diuretics (65 (92.9) vs. 67 (95.7) vs. 59 (84.3); *p* = .049), beta blockers (60 (85.7) vs. 58 (82.9) vs. 43 (61.4); *p* = .001), ACEI/ARB (50 (71.4) vs. 54 (78.3) vs. 40 (57.1); *p* = .05) than HFpEF groups. There were no significant differences in the use of spironolactone (56 (80.0) vs. 61 (87.1) vs. 64 (91.4); *p* = .141), digoxin (6 (8.6) vs. 11 (15.7) vs. 11 (15.7); *p* = .357), anticoagulants (32 (57.5) vs. 27 (38.6) vs. 34 (48.6); *p* = .471), and statins (54 (77.1) vs. 47 (67.1) vs. 47 (68.1); *p* = .358) among HFpEF, HFmrEF, and HFrEF groups.

The summary related to the clinical characteristics of the three groups is shown in Table 1.

**Table 1:** Baseline data of patients.

	HFpEF (70)	HFmrEF (70)	HFrEF (70)	P1‐2	P2‐3	P1‐3
Age (years old)	77, 8.75	73, 8.00	74, 7.00	0.146		
Male, number of cases (%)	32 (46)	39 (56)	45 (64)	0.087		
Smoking, number of cases (%)	19 (27.1)	26 (37.1)	15 (21.4)	0.114		
Drinking alcohol, number of cases (%)	13 (18.6)	17 (24.3)	8 (11.4)	0.141		
Hypertension, number of cases (%)	52 (74.3)	40 (57.1)	25 (35.7)	*p* < .001		
Diabetes mellitus, number of cases (%)	25 (35.7)	15 (21.4)	18 (25.7)	0.152		
*Previous medical history*						
Atrial fibrillation, number of cases (%)	43 (61.4)	32 (45.7)	38 (54.3)	0.175		
Mild or moderate mitral regurgitation, number of cases (%)	49 (70.0)	55 (78.6)	56 (80.0)	0.323		
Mild or moderate mitral stenosis, number of cases (%)	5 (7.1)	5 (7.1)	7 (10.0)	0.774		
*Drugs*						
Diuretics, number of cases (%)	59 (84.3)	65 (92.9)	67 (95.7)	0.049		
Spironolactone, number of cases (%)	56 (80.0)	61 (87.1)	64 (91.4)	0.141		
Digoxin, number of cases (%)	6 (8.6)	11 (15.7)	11 (15.7)	0.357		
Anticoagulants, number of cases (%)	32 (57.5)	27 (38.6)	34 (48.6)	0.471		
Statins, number of cases (%)	54 (77.1)	47 (67.1)	47 (68.1)	0.358		
B resistance agent, number of cases (%)	43 (61.4)	60 (85.7)	58 (82.9)	0.001		
ACEI/ARB, number of cases (%)	40 (57.1)	50 (71.4)	54 (78.3)	0.05		
Laboratory index	547.50, 1160.25	1091.50, 1652.75	1584.67, 2481.75	0.021	0.128	<0.001
BNP	36.25, 4.35	35.10, 5.93	35.70, 5.70	0.313	0.007	0.007
Alb (g/L)	77.5, 36.75	80, 35.25	100, 46.25	1.0		
Cr (mmol/L)	0.86, 0.61	0.92, 0.62	0.93, 0.52	0.944		
TG (mmol/L)	3.42, 1.28	3.60, 1.13	3.47, 1.33	0.476		
TC (mmol/L)	1.89, 1.02	2.33, 0.75	2.02, 1.02	0.002	0.054	0.529
LDL‐C (mmol/L)	1.10, 0.43	0.99, 0.45	1.06, 0.38	0.012	1.0	0.447
HDL‐C (mmol/L)	11.85, 5.88	13.25, 6.13	16.5, 6.55	0.876	0.038	0.006
HCY (mmol/L)						
Cardiac ultrasound	0.63, 0.11	0.42, 0.04		<0.001	<0.001	<0.001
LVEF (%)	40, 11	44, 13	0.34, 0.06	0.052	1.0	0.007
LAD (mm)	48, 8.0	56, 9.0	44.5, 9.25	<0.001	<0.001	<0.001
LVEDd (mm)	32, 8.0	42, 9.25	64, 11.25	<0.001	<0.001	<0.001
LVEDs (mm)	11.4, 2.53	11.2, 3.13	54, 12	1.0	0.002	0.001
IVSd (mm)	10.55, 2.5	10.2, 1.85	10.15, 2.23	0.202		
LVPWd (mm)			9.6, 1.4			

Abbreviations: BNP, B‐type brain natriuretic peptide; Cr, creatinine; HCY, homocysteine; HDL‐C, high‐density lipoprotein; IVSd, interventricular septum thickness at end‐diastolic; LAD, Left atrial diameter; LDL‐C, low‐density lipoprotein; LVEDd, left ventricular end‐diastolic diameter; LVEDs, left ventricular end‐systolic diameter; LVEF, left ventricular ejection fraction; LVPWD, left ventricular posterior wall diastolic thickness; TG, triglyceride; TT, total cholesterol.

### 3.2 ROC curve of LAD and atrial fibrillation

**Figure 1:**
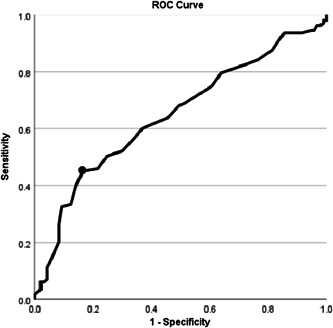
The receiver operating characteristic curve of left atrial diameter and atrial fibrillation in patients with heart failure. The circled point is the best cut‐off point. Youden index = 0.29, sensitivity = 45.1%, specificity =  83.50%. The area under the curve (AUC) = 0.65 (95% CI: 0.57–0.72) (*p* < .001).

ROC analysis revealed that the optimal cutoff value of the LAD for predicting the prevalence of atrial fibrillation in patients with heart failure was 0.286 (AUC: 0.65; 95% CI: 0.57–0.72; *p* < .001; Figure 1). The sensitivity and specificity were 45.1% and 83.5%, respectively.

### 3.3 Correlation between LAD and related indexes of heart failure

There was a negative correlation between LAD and LVEF (correlation coefficient: −0.209; *p* = .002), a positive correlation between LAD and LVEDd (correlation coefficient: 0.299; *p* < .0021), and no correlation between LAD and BNP and NYHA grades (*p* = .308 and *p* = .272).

### 3.4 Difference of LAD and BNP in HFpEF, HFmrEF, and HFrEF

The LAD of HFrEF group was higher than that of HFpEF group (44.5, 9.25 vs. 40.00, 11.00), there was no significant difference in LAD between HFrEF group and HFmrEF group (44.5, 9.25 vs. 44.00, 13.00), and there was no significant difference in LAD between HFpEF group and HFmrEF group (40.00, 11.00 vs. 44.00, 13.00).

### 3.5 ROC curve of LAD and HFrEF

**Figure 2:**
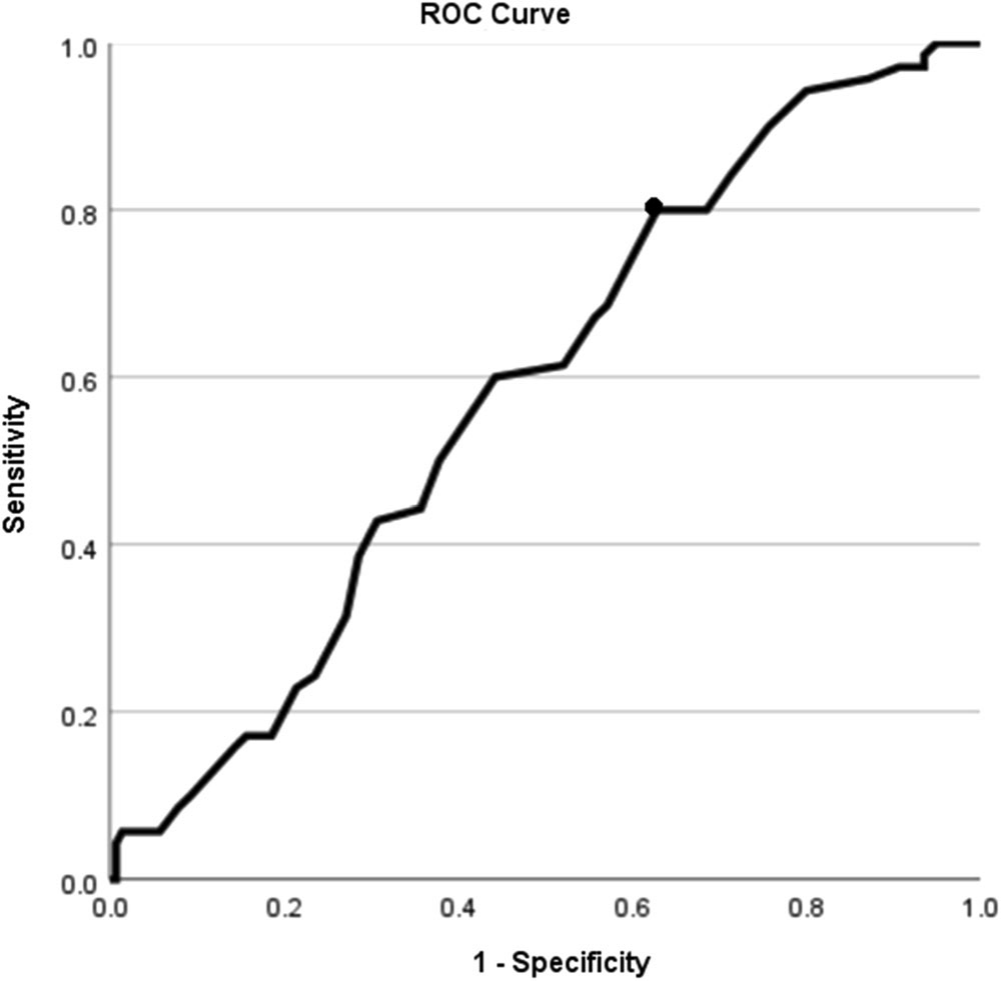
The receiver operating characteristic curve of left atrial diameter and heart failure reduced ejection fraction in patients with heart failure. The circled point is the best cut‐off point. Youden index = 0.17, sensitivity = 80.0%, specificity = 37.1%. The area under the curve (AUC) = 0.58 (95% CI: 0.51–0.66) (*p* = .047).

ROC analysis revealed that the optimal cutoff value of the LAD for predicting the prevalence of HFrEF was 0.17 (AUC: 0.58; 95% CI: 0.51–0.66; *p* = .047; Figure 2). The sensitivity and specificity were 80.0% and 37.1%, respectively.

## 4 DISCUSSION

In this retrospective study, a total of 210 patients with heart failure were included. According to the new diagnostic criteria^1^ in ESC guidelines in 2016, heart failure was divided into three groups according to ejection fraction: HFpEF, HFmrEF, and HFrEF. There was no significant difference in age, sex, smoking history, drinking history, diabetes history and atrial fibrillation among the three groups (*p* = .146, *p* = .087, *p* = .114, *p* = .141, *p* = .152, and *p* = .175).

The comparison of basic diseases showed that HFrEF had the lowest proportion of hypertension patients (35.7%) and HFpEF had the highest proportion of hypertension patients (74.3%), with statistical difference (*p* < .001). Hypertension plays an important role in pathological left ventricular hypertrophy, which inducing process of heart failure.^17^

In this study, with the decrease of LVEF, NT‐proBNP gradually increased, but the difference between the three groups was not statistically significant (P1 = 0.021; P2 = 0.128; P3 ≤ 0.001). Previous studies suggest that there may not be a clear BNP threshold that can effectively distinguish HFpEF from HFrEF.^18^

From the perspective of left ventricular structure and function, LVEDd and LVEDs in HFrEF group is the largest, and IVSd in HFpEF group is the thickest, suggesting that HFrEF is characterized by left ventricular enlargement and systolic dysfunction, while HFpEF is mainly characterized by left ventricular concentric remodeling/hypertrophy and diastolic dysfunction, which is similar to previous studies.^19^, ^20^ Left atrial enlargement is a reflection of physiological disorder of heart disease, which suggests that left ventricular filling pressure is increased, volume and pressure load are overloaded, which increases the risk of atrial fibrillation, stroke and heart failure in the population, and can predict the death risk of high‐risk groups (dilated cardiomyopathy, atrial arrhythmia, acute myocardial infarction, aortic valve replacement, etc.).^21^ More and more studies have confirmed that left atrial enlargement is a risk factor for poor prognosis in patients with heart failure, As the meta‐analysis results of Rossi et al. show, with the increase of left atrial enlargement, death or rehospitalization risk of heart failure, the incidence of end events in patients with larger left atrial area (≥median) is 1.4 times that in patients with smaller left atrial area (<median), and the incidence of end events in patients with larger left atrial area index is 2.36 times that in patients with smaller left atrial area index.^14^ Another meta‐analysis involving 1188 patients with heart failure showed that with the increase of left atrial volume index (LAVI), the risk of ACD increased.^22^

This study shows that with the decrease of systolic function, the left atrium gradually expands, namely the lower LVEF is, the larger LAD is. There is a significant difference between HFpEF and HFrEF group in LAD (*p* = .007), and LAD is negatively correlated with LVEF (*p* = .002, *r* = −.209). Therefore, it is speculated that LAD may be used to predict the occurrence of HFrEF.

It is found that LAD has high sensitivity (sensitivity for 80.0%) in predicting HFrEF, because left atrial enlargement is an important pathogenic mechanism of left atrial remodeling. Because left atrial enlargement reflects chronic left ventricular end‐diastolic pressure increase, patients with heart failure with left atrial enlargement tend to have longer course of disease, worse cardiac function and more serious cardiac remodeling.^23^ However, the specificity of LAD in predicting HFrEF is low (specificity is 37.1%). Previous studies have also confirmed that LAD size is related to heart failure,^24^ hypertension,^25^ myocardial ischemia,^26^ obesity,^27^ and obstructive sleep apnea,^28^ which is consistent with our expectations.

This study is a single‐center retrospective study, which has the inherent limitations of retrospective study and a small sample size. Second, all patients excluded AF/AFL through symptoms, signs, past history, physical examination, past and postadmission ordinary and 48 h ambulatory electrocardiogram, which may ignore the possibility of occult AF, and the results may be biased. In the future, a larger and multicenter cohort study is needed to verify and support it. In addition, we failed to obtain the data of left atrial volume in this study. Although LAD has a strong correlation with left atrial volume,^29^ it is recommended to use left atrial volume to evaluate left atrial size at present.^30^ This difference may affect the universality of our results.

## 5 CONCLUSION

LAD is negatively correlated with LVEF, which may predict the occurrence of prevalent HFrEF.

## CONFLICT OF INTEREST STATEMENT

The authors declare no conflict of interest.

## ETHICS STATEMENT

This research was conducted following the Declaration of Helsinki. This retrospective study was approved by the Institutional Review Board of Ningbo medical center lihuili hospital and waived the individual consent for this analysis.

## Data Availability

The datasets generated and analyzed during the current study are available from the corresponding author on reasonable request.
